# Myositis of pterygoid muscles and superior ophthalmic vein and cavernous sinus thrombosis in active Crohn’s disease undergoing ustekinumab treatment: a case report and literature review

**DOI:** 10.3389/fphar.2025.1544466

**Published:** 2025-02-26

**Authors:** Robbe Winters, Sara Kaut, Dries Govaerts, Aaron De Poortere, Ilse Mombaerts, Ilse Hoffman, Robin Willaert, Karen van Hoeve

**Affiliations:** ^1^ Department of Oral and Maxillofacial Surgery, University Hospitals Leuven, Leuven, Belgium; ^2^ Department of Pediatric Gastroenterology and Hepatology and nutrition, University Hospitals Leuven, KU Leuven, Leuven, Belgium; ^3^ OMFS-IMPATH Research Group Department of Imaging and Pathology, Faculty of Medicine, KU Leuven, Leuven, Belgium; ^4^ Department of Ophthalmology, University hospitals Leuven, Leuven, Belgium; ^5^ Department of Neurosciences, KU Leuven, Leuven, Belgium

**Keywords:** inflammatory bowel diseases, myositis, ustekinumab, pediatrics, case report

## Abstract

**Introduction:**

Myositis is a rare extra-intestinal presentation in patients with inflammatory bowel diseases (IBD), and its occurrence has only been described in a few case reports. However, it is essential to consider other potential causes as patients with IBD are more susceptible to infections due to their immunocompromised status, which may also be exacerbated by immunosuppressive drugs contributing to myositis. Our case highlights the complexity and challenges in diagnosing and managing myositis in patients with IBD as well as a review of the literature.

**Case report:**

We report the case of a 17-year-old girl with active Crohn’s disease (CD) undergoing ustekinumab (UST) treatment. She presented with sudden onset of pain and rapid progressive swelling of both jaws, along with eyelid swelling, blurred vision, and diplopia. Radiologic imaging revealed myositis affecting the pterygoid muscles, combined with thrombosis of the left superior ophthalmic vein and (partially) of the left cavernous sinus. Symptoms resolved completely after stopping UST treatment and initiating corticosteroids and enoxaparine.

**Discussion:**

Our report accounts for the second patient described in literature with myositis associated with CD while being treated with UST. The causal factor remains speculative, as both active CD and UST treatment may contribute to this complication. Sharing clinical experiences and reporting possible adverse events to regulatory agencies can enhance our understanding of rare complications and improve patient outcomes by providing therapeutic strategies.

## Introduction

Myositis is a condition characterized by inflammation of skeletal muscle tissue and can be triggered by various underlying factors. Aside from the more common infectious myositis, it is essential to consider other potential causes such as metabolic myopathy, endocrinopathy, neuromuscular disorders, and immune mediated diseases (Rendt, 2001). These latter encompass not only the well-known idiopathic inflammatory myopathies like polymyositis, dermatomyositis, and inclusion body myositis but also other systemic inflammatory conditions, like inflammatory bowel diseases (IBDs) (Rendt, 2001).

IBDs are heterogeneous diseases primarily affecting the gastrointestinal tract, typically classified as Crohn’s disease (CD) and ulcerative colitis (Torres et al., 2017; Ungaro et al., 2017). However, IBD can also present with extra-intestinal manifestations (EIMs) in approximately 25%–40% of patients. Pediatric IBD patients without an EIM at time of diagnosis will develop in 29% of the cases at least one EIM within 15 years after diagnosis (Jose et al., 2009). Given that EIMs affect morbidity and sometimes even mortality, rapid diagnosis and adequate treatment are necessary. EIMs can involve various organs, with the skin, joints, biliary tract, and eyes being most affected. Less frequently, EIMs may also impact other organ systems, including the muscles, leading to myositis (Harbord et al., 2016).

While the management of EIMs primarily revolves around treating the underlying intestinal disease, it's crucial to acknowledge that myositis can also be a side effect of numerous drugs. Especially drugs such as erythromycin and ketoconazole, which inhibit cytochrome P450 3A4 (Rendt, 2001), but also immunosuppressive drugs used to treat IBD can contribute to myositis, making it challenging to differentiate between myositis caused by EIM or drug-induced myositis (Zhou et al., 2015; Gaboriau et al., 2020; Yoshida et al., 2021; Jordan et al., 2022; Zengin et al., 2017; Chavarría-Miranda et al., 2021). Furthermore, IBD patients are more susceptible to infections due to their immunocompromised status, which could further contribute to myositis (Gong et al., 2019). These complexities necessitate a comprehensive, multidisciplinary approach to establish an appropriate treatment plan.

In this context, we present a rare case of myositis affecting masticatory muscles complicated with cavernous sinus and superior ophthalmic thrombosis in a young girl with active CD during a treatment with ustekinumab (UST). UST is a fully human monoclonal antibody targeting the p40 subunit of interleukin (IL)-12 and IL-23. Its efficacy in treating IBD has been demonstrated in pivotal phase 3 trials, UNITI for CD and UNIFI for ulcerative colitis, both of which reported a favorable safety profile (Sands et al., 2019; Sandborn et al., 2012). Commonly reported side effects include nausea, abdominal pain, rash, headache, arthralgia, injection-site reactions, and upper respiratory infections (Honap et al., 2022). However, ongoing post-marketing pharmacovigilance is crucial to identify less frequent or rare adverse events. This is, to the best of our knowledge, the second reported case in the English literature of myositis associated with CD during UST treatment. A thorough search across multiple databases using terms such as “Ustekinumab,” “Stelara,” and “myositis” underscores the rarity of this condition in combination with IBD and UST treatment (Smith et al., 2021; Ezekwe et al., 2021; Vasudevan et al., 2022). While this phenomenon does not appear to be class-specific—given that drug-induced myositis has also been reported infrequently with anti-tumor necrosis factor (TNF) alpha agents and rarely with vedolizumab—the precise role of IL-12/IL-23 inhibition in triggering myositis remains unclear (Zhou et al., 2015; Gaboriau et al., 2020; Yoshida et al., 2021; Jordan et al., 2022; Zengin et al., 2017; Chavarría-Miranda et al., 2021). These findings highlight the importance of further investigation into the underlying mechanisms to better understand this rare complication.

## Case report

A 17-year-old girl had a 2-year history of CD. She was a primary non-responder to TNF alpha therapy and was subsequently switched to UST on an eight-weekly injection schedule of 90 mg, which was later increased to four-weekly due to loss of response after 7 months. Clinical remission was achieved under this intensified treatment, but 6 months later, moderate endoscopic disease activity persisted, leading to a planned switch to intravenous vedolizumab upon her 18th birthday. The patient, otherwise healthy, recently started oral contraceptive therapy.

Around the 11th month of four-weekly UST administration, just before the planned switch, she experienced acute onset of pain and swelling in the right jaw, which progressively worsened throughout the day. Her medical history revealed no head and neck pathology or recent infectious diseases, and regular annual dental check-up showed no significant findings. Clinical examination demonstrated painful palpation and swelling of the right jaw with restricted mouth opening (15–20 mm). There was no pain or discomfort in the temporomandibular joint. Intra-oral examination revealed swelling of the maxillary vestibule in the first quadrant, sensitive on palpation, with no dental focus. Palpation of the right parotid gland was painful, but saliva production remained normal.

**FIGURE 1:**
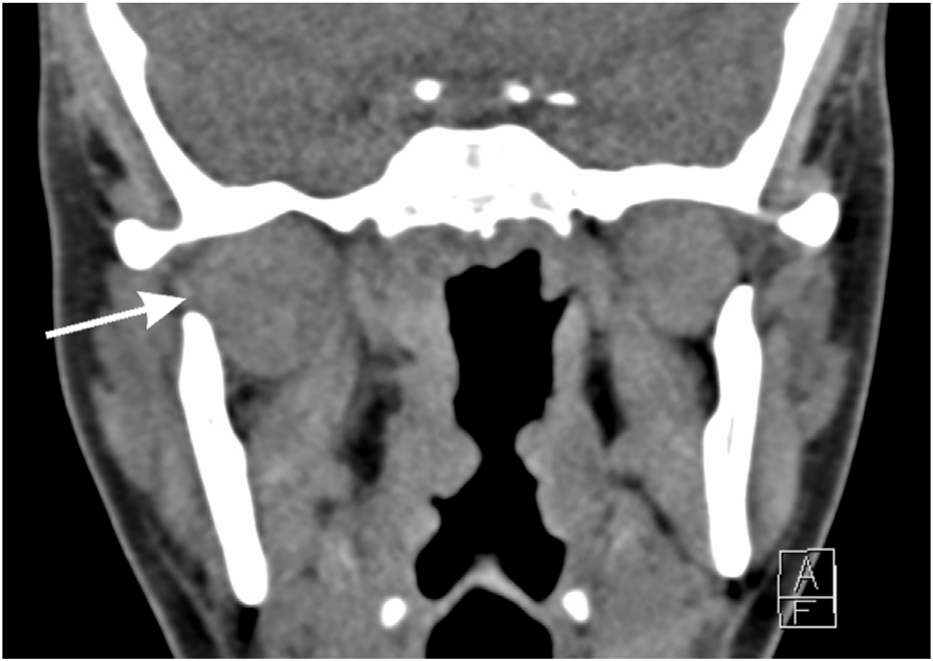
Computed tomography scanning of the head and neck. Coronal view showing enlargement of the right lateral pterygoid muscle and surrounding inflammation as well as fat infiltration, indicated with the arrow.

Panoramic radiographical scanning showed no abnormalities. Ultrasound of the jaw revealed inflammation of the buccal fat adjacent to the right parotid gland, without evidence of parotitis. Head and neck computed tomography scanning disclosed diffuse enlargement of the right lateral pterygoid muscle with fat infiltration in the parapharyngeal space (Figure 1). Dental and temporomandibular joint pathology were ruled out. Blood test showed slight elevation in C-reactive protein (10 mg/L) and erythrocyte sedimentation rate (18 mm/h), indicating active CD. Creatinine kinase levels were normal and serology for Epstein-Barr virus was negative. Respiratory panel was negative for 29 respiratory micro-organisms from a nasopharyngeal swab. Clinical and radiological features were compatible with myositis of the lateral pterygoid muscle. An initial treatment plan included celecoxib (200 mg twice daily), painkillers (including tramadol), and intravenous amoxicillin-clavulanic acid.

**FIGURE 2:**
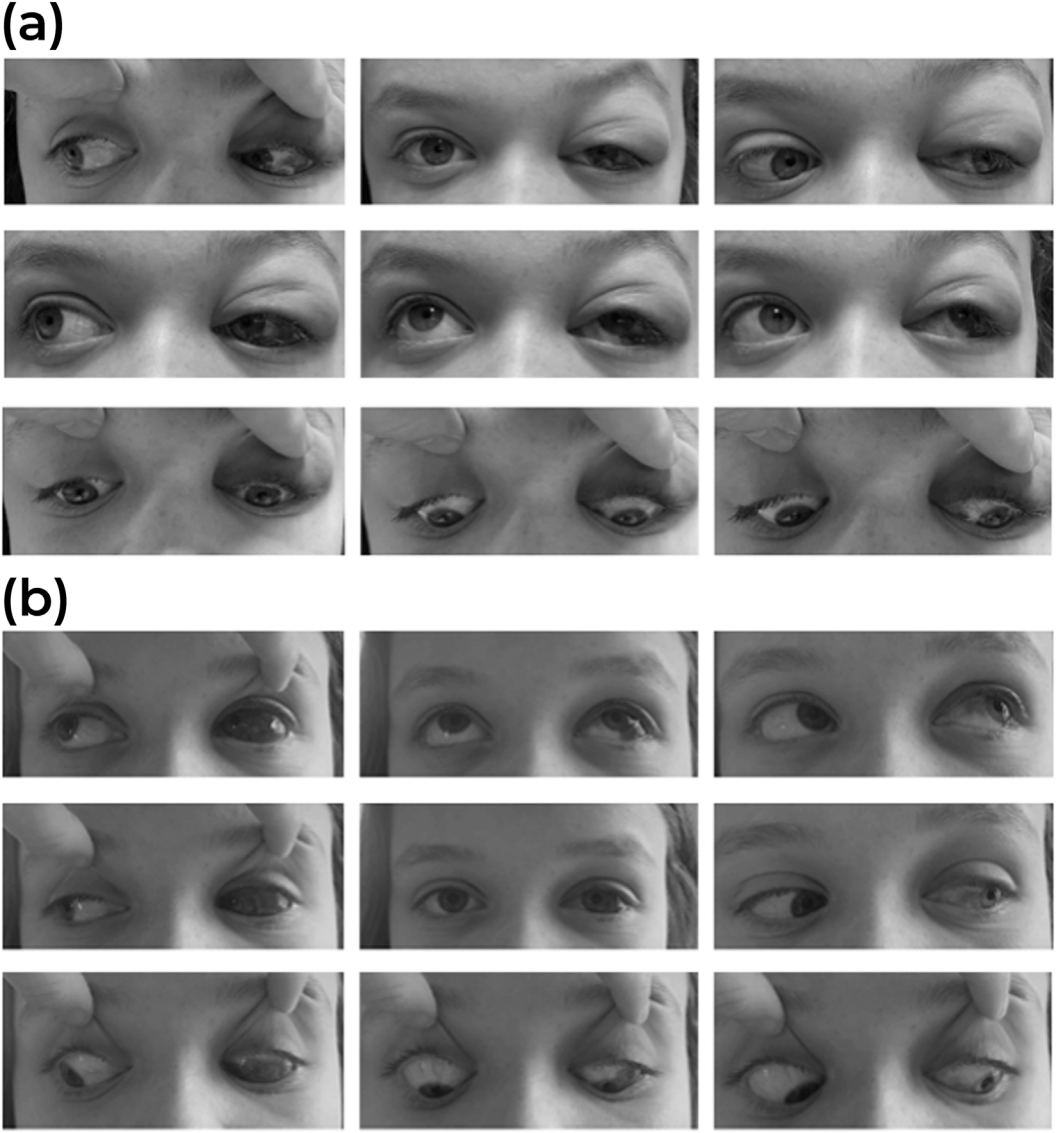
**(A)** Nine gaze clinical exam. Peri-orbital swelling and ecchymosis of the left upper eyelid with bulbar conjunctival chemosis and hemorrhage at the lateral aspect of the left eye. Elevation and abduction of the left eye are mildly impaired. **(B)** Same exam, 1 day after the start of the steroid treatment. Improvement of the eyelid swelling and the eye motility.

**FIGURE 3:**
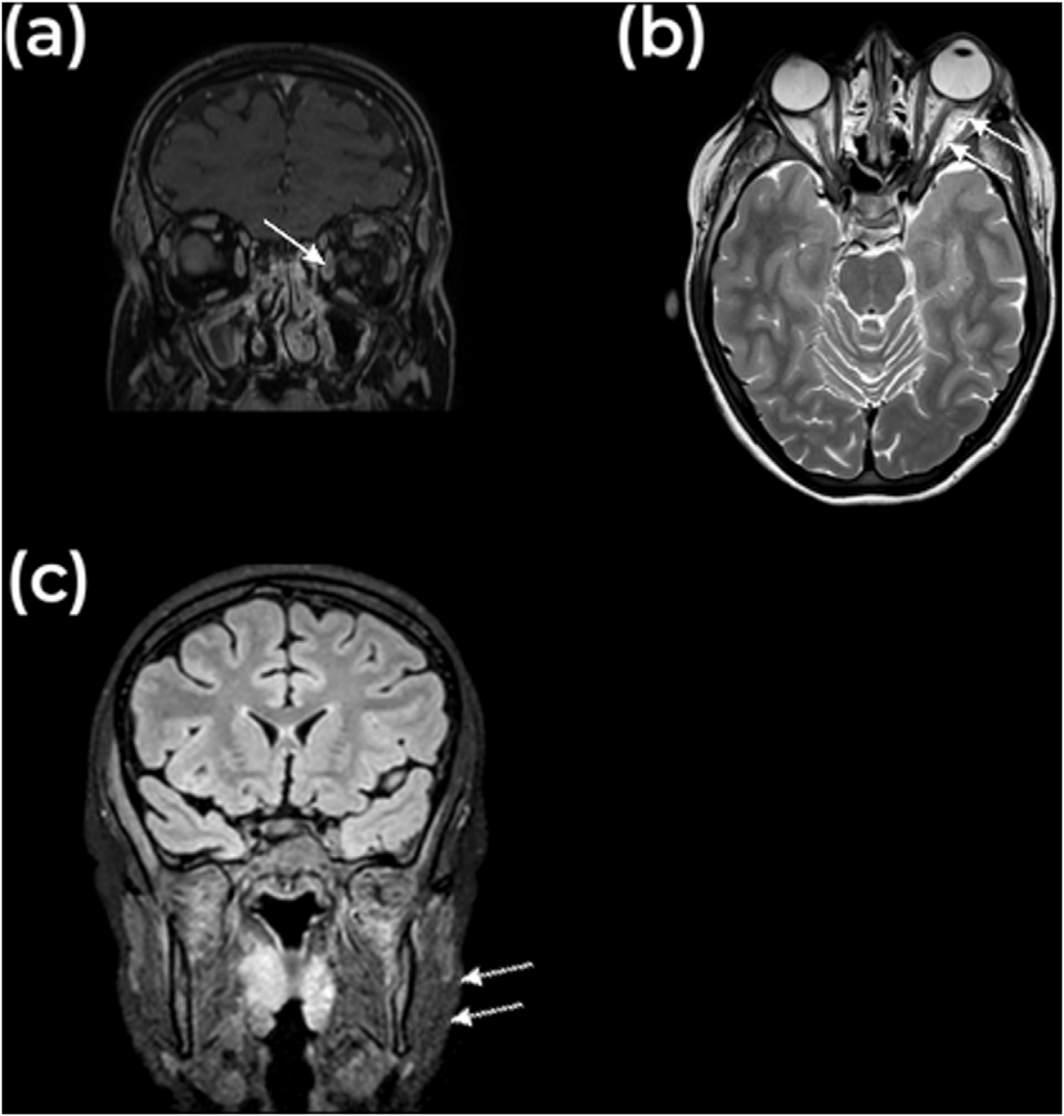
MRI findings. **(A)** Coronal and **(B)** Axial view of MRI image, T1 weighted, showing axial proptosis with infiltration of the intraconal fat and a non-perfused enlarged superior ophthalmic vein, indicated with the arrow. The superior lateral and medial rectus muscles are slightly enlarged. **(C)** Coronal view, showing enlargement of the masticator muscles, indicated with the arrow.

However, within 24 h, she experienced rapidly progressive swelling in the contralateral, left maxillary region, and peri-orbital soft tissues (Figure 2). Blurred vision and diplopia were reported. Ophthalmological examination showed proptosis, mildly limited eye motility in elevation and abduction, increased intraocular tension, and conjunctival bulbar chemosis and ecchymosis in the lateral sector of the eye. A new and more detailed radiographic evaluation using magnetic resonance imaging (MRI) was deemed necessary to obtain high-resolution images without subjecting the patient to additional radiation. This was particularly important, as a computed tomography scan had been used at the time of initial presentation due to the unavailability of MRI. The MRI revealed bilateral myositis of medial and temporal pterygoid muscle, along with inflammation extending to the pterygopalatine fossa and partial thrombosis of the left cavernous sinus and total thrombosis of the left superior ophthalmic vein. Additionally, diffuse fat infiltration of the left orbit, proptosis, stretching of the optic nerve, and mild enlargement of the left superior and lateral rectus muscles were evident (Figure 3).

Treatment involved discontinuation of UST and administering subcutaneous enoxaparin (1 mg/kg twice daily), oral celecoxib (200 mg twice daily), and intravenous prednisolone (1 mg/kg per day) to counteract rapid progression. Remarkably, within 72 h, orbital inflammation dramatically improved, with normalization of intraocular tension and movement. Jaw swelling and pain resolved in the following 3 days. A follow-up MRI 3 months later showed complete resolution of the condition, including the thrombosis. As planned, 1 month later, she was switched to intravenous vedolizumab to manage her CD effectively. Therapeutic enoxaparine was given for 3 months. No relapse was observed at the 9-month follow-up. This work was carried out in accordance with The Code of Ethics of the World Medical Association (Declaration of Helsinki). Formal ethical approval was obtained (S70245). Written informed consent was obtained from the patient and legal guardians for the publication of this case report, including any potentially identifiable images or data included in this article.

## Discussion

Many factors may contribute to myositis development. In our case, the patient presented with active CD while being treated with UST, which complicates identifying the specific causative factor of the myositis.

Myositis is an infrequent EIM of IBD, primarily documented through case reports, and more commonly associated with acute flare-ups of CD rather than ulcerative colitis (Bourikas and Papadakis, 2009). Patients often experience acute onset of pain and swelling in different muscle regions, even with mild IBD activity. Among reported cases, 34 patients had facial muscle myositis (See Supplementary Table S1). In particular, CD-associated orbital myositis, usually affecting one or more horizontal rectus muscles, is documented (McNab, 2020). Diagnosis often involves MRI revealing muscle enlargement and a high-intensity signal indicative of inflammation (McNab, 2020). High-dose corticosteroid therapy is frequently employed due to its potential to prevent permanent visual damage if left untreated. Often leading to rapid symptom reduction and complete recovery (McNab, 2020; Culver et al., 2008; Sandhu et al., 2022; Robertson et al., 2022). However, treatment decisions lack definitive evidence, necessitating individualized management, as some patients may relapse upon corticosteroid tapering or discontinuation (Sandhu et al., 2022; Pimentel et al., 2012).

Interestingly, a small number of patients with UST-induced myositis have been disclosed. UST (Stelara^®^ by Janssen-Cilag international BV, Beerse, Belgium) is as a monoclonal antibody targeting p40 protein subunit of both interleukin-12 and 23, which are pro-inflammatory cytokines that have been implicated in the pathogenesis of IBD (Sands et al., 2019; Sandborn et al., 2012). It induces and maintains remission in adult IBD patients (Sands et al., 2019; Sandborn et al., 2012) with a favorable safety profile (Honap et al., 2022). Our literature search revealed three cases of myositis associated with UST treatment, two of which involved off-label use for hidradenitis suppurativa (Smith et al., 2021; Ezekwe et al., 2021; Vasudevan et al., 2022). Myositis often manifested shortly after starting UST in these patients, and symptoms varied, including muscle weakness and localized pain in non-specific muscles (bicep, gastrocnemius, extensor carpi radialis, lateral and medial rectus muscles) (Smith et al., 2021; Ezekwe et al., 2021; Vasudevan et al., 2022). Laboratory exams for creatine phosphokinase levels were not diagnostic as more pronounced elevation occurs only with expansive muscle degradation. Discontinuation of UST resolved symptoms in hidradenitis suppurativa cases, while the CD patient needed corticosteroids for orbital myositis (Smith et al., 2021; Ezekwe et al., 2021; Vasudevan et al., 2022). Moreover, 13 cases were reported to the European Medicines Agency and 22 to the American Food & Drug Administration (Oracle BI Interactive Dashboards - NTS, 2018; The Food and Drug Administration, 2022). Drug-induced myositis has also been rarely described after anti-TNF alpha treatment (to the best of the authors’ knowledge 58 cases have been found throughout literature), suggesting a non-class-specific phenomenon (Zhou et al., 2015; Gaboriau et al., 2020; Yoshida et al., 2021; Jordan et al., 2022; Zengin et al., 2017; Chavarría-Miranda et al., 2021; Mukharesh and Tinsley, 2022; Kato et al., 2014; Caramaschi et al., 2003). To date, there are no documented cases of myositis associated with other IL-23 inhibitors, such as risankizumab, guselkumab, or mirikizumab. However, it is important to note that these IL-23 inhibitors have only recently been approved by regulatory agencies. As such, it is possible that this rare side effect has not yet been observed due to the limited time these treatments have been on the market. The exact role of IL-12/IL-23 inhibition in the onset of myositis remains unclear, highlighting the need for further investigation into its underlying mechanisms.

In our patient’s case, high-dose corticosteroids were administered alongside discontinuation of UST to ensure rapid regression of jaw and orbital symptoms. Considering the swift resolution, both EIM and UST-induced myositis remain possible causes. This is essential for the follow-up, as recurrent episodes of acute myositis may occur (Bourikas and Papadakis, 2009; Sandhu et al., 2022; Pimentel et al., 2012).

While a definitive link has not been established, rare adverse events in pediatric populations are challenging to identify early due to their low prevalence and nonspecific presentation. This often delays appropriate interventions and presents unique challenges that influence clinical decisions, patient outcomes, and long-term care strategies. The need for robust pharmacovigilance systems in pediatric biologic therapy is critical. Reporting rare adverse events is essential for building a more comprehensive safety profile, improving risk assessments, and guiding safer prescribing practices. Especially since there is a significant lack of well-established pharmacokinetic data for many drugs and biologics in pediatric populations. Recognizing this gap, the Food and Drug Administration published in 1998 an act recommending that pediatric pharmacokinetic studies evaluate appropriate medication doses across all age groups, from neonates to adolescents, to achieve systemic exposure levels like those considered safe and effective in adults (The Food and Drug Administration, 1998). This is particularly important in pediatrics because growth and developmental changes influence drug absorption, distribution, metabolism, and excretion. Real-world data can help address the limitations of clinical trials, which often have insufficient sample sizes for pediatric populations, restricting our understanding of long-term safety profiles in younger patients.

Our case was complicated with partial thrombosis of the cavernous sinus and superior ophthalmic vein, likely due to a combination of factors: active CD, local inflammation from pterygoid myositis, and the pro-thrombotic effect of recently started oral contraceptive therapy (Bollen et al., 2016). Therapeutic enoxaparin, with both anti-coagulant and anti-inflammatory properties, was prescribed for 3 months to address this. After achieving full recovery, the medication was discontinued. This underscores the need for vigilant monitoring and thromboprophylaxis in IBD patients with active disease.

In conclusion, our case highlights the complexity of diagnosing and managing myositis in active CD patients undergoing UST treatment. The presentation of facial muscle myositis further emphasizes the importance of considering EIM of CD. Additionally, occurrence of UST-induced myositis adds to the therapy’s diverse side effects. Urgent treatment with high-dose corticosteroid, discontinuation of UST, and a switch to intravenous vedolizumab for effective management of her CD were crucial for a complete remission. No relapse was observed at the 9-month follow-up. This case underscores the importance of early recognition, prompt intervention, and a collaborative approach in managing myositis in the context of CD and its treatment. Ongoing vigilance and a comprehensive understanding of the complex interactions between the disease, its treatment, and potential complications are essential to optimize patient care and achieve favorable outcomes.

## Data Availability

The original contributions presented in the study are included in the article/Supplementary Material, further inquiries can be directed to the corresponding author.

## Abbreviations

CD, Crohn’s disease; EIM, extra-intestinal manifestation; IBD, inflammatory bowel disease; IL, interleukin; MRI, magnetic resonance imaging; TNF, tumor Necrosis Factor; UST, Ustekinumab.
