# Supplementary material for: Myositis of pterygoid muscles and superior ophthalmic vein and cavernous sinus thrombosis in active Crohn’s disease undergoing ustekinumab treatment: a case report and literature review
Source: Front Pharmacol. 2025 Feb 26;16:1544466. doi: 10.3389/fphar.2025.1544466 (PMC11897002; doi:10.3389/fphar.2025.1544466)
Supplement: Supplementary file 1 [file Table1.docx]

**Supplementary table 1.** Published case reports describing patients with inflammatory bowel disease (IBD) and myositis of the head-neck region.

| **No** | **Age (yr.)** | **Sex** | **IBD type** | **Time to presentation** | **Muscle localization** | **Previous therapy** | **Therapy adapted to** | **Outcome**  **(total FU time)** | **No. (Ref)** |
| --- | --- | --- | --- | --- | --- | --- | --- | --- | --- |
| 1 | 63 | M | UC | +9 mo. | Bilateral, all extra-ocular muscles | IFX (prior: ASA) | CS and optimization IFX | Rapid improvement after 72h., complete resolution after 2 wk. No relapse over 13 mo. (22 mo.) | [1] |
| 2 | 35 | F | CD | -1 yr. and at diagnosis | 1^st^ event: NM  2^nd^ event: MRL sinister | NON | 1^st^ event: CS  2^nd^ event: CS, AZA, and SSZ | Excellent response (NM). No relapse over 1 yr. (2 yr.) | [2] |
| 3 | 15 | F | CD | -1 mo. | Non-specified extra-ocular muscles | NON | CS | Rapid improvement after 24h., complete resolution after 1 mo. No relapse over 3 mo. on CS. (4 mo.) | [3] |
| 4 | 38 | F | CD | -2 yr., +6 yr, and +6 yr. 3 mo. | 1^st^ event: NM  2^nd^ event: MRM and MRI sinister  3^rd^ event: MRI sinister and MRM dexter | 1^st^ event: NON  2^nd^ and 3^rd^ event:  ICR, 6-MP, and redo- ICR | 1^st^ event: NM  2^nd^ event: CS  3^rd^ event: CS and increase of 6-MP | 2^nd^ and 3^rd^ event rapid improvement after 48h. But relapse 3 mo. after 2^nd^ event and no data after 3^rd^ event (13 yr.) | [4] |
| 5 | 30 | F | CD | +7 yr. | MRL and MRM sinister | IFX (prior: ICR and MTX) | CS, stop IFX, and start CP  Later switch to ADA due to active CD (start after resolution myositis). | No effect of CS and IFX, resolution after 2^nd^ dose of CP without relapses over 9 mo. (8 yr.) | [5] |
| 6 | 12 | F | CD | -8 mo., -5 mo., and at diagnosis | 1^st^ event: NM  2^nd^ event: orbital muscles,  3^rd^ event: bilateral MRL, MRM, and MRI | NON | 1^st^ event: NON  2^nd^ event: NON  3^rd^ event: CS and ASA | Rapid improvement after 24h of start of CS, with no relapse over 6 mo. on ASA (14 mo.) | [6] |
| 7 | 34 | F | CD | NM | 1^st^ event: MRM  multiple relapses over 4 yr.: NM | NM | CS (repeatedly), MTX, ICR, and IFX | Only recovered after IFX, with no relapses over 2 yr. (7 yr.) | [7] |
| 8 | 33 | F | CD | -2 yr. until +4 yr. | MRL | 1^st^ event: NON | CS, MTX, and radiotherapy, prior to diagnosis of CD.  At diagnosis: colectomy and MTX. 6-MP 1 yr. after, and IFX 4 yr. after diagnosis. | Improvement after colectomy  Only recovered after IFX, with no relapses over 27 mo. (8 yrs.) | [7] |
| 9 | 32 | F | CD | -2 yr. and at diagnosis | Bilateral MRM and MRS | NON | 1^st^ event: CS and add-on CsA due to CS dependence  2^nd^ event: stop CsA and start ADA | Only remission after start of ADA, no relapse over 3 yr. (5 yr.) | [8] |
| 10 | 28 | F | CD | -10 mo. and at diagnosis | 1^st^ event: left MRM  2^nd^ event: NM | NON | 1^st^ event: CS  2^nd^ event: CS | Improvement after first event, no outcome mentioned after second event (10 mo.). | [9] |
| 11 | 9 | M | CD | -18 mo., -17 mo., -7 mo., and at diagnosis | 1^st^ event: MRL and MRM sinister  2^nd^ and 4^th^ event: NM  3^rd^ event: MRS and MRI dexter | 1^st^, 2^nd^, and 3^rd^ event: NON  4^th^ event: MTX | 1^st^ and 2^nd^ event: CS  3^rd^ event: CS and MTX  4^th^ event: ADA | After 3^rd^ event: residual CN3 palsy and intraocular HTN OD. Remission after start of ADA, no relapse over 2 mo. (2 yr.) | [10] |
| 12 | 14 | F | UC | At diagnosis | Bilateral masseter muscles | NON | CS and SSZ | Rapid improvement, no relapses over 1 yr. (1 yr.) | [11] |
| 13 | 43 | F | UC | +7 yr. and +12 yr. | 1^st^ event: MRM sinister  2^nd^ event: MRL dexter | NON | 1^st^ event: CS  2^nd^ event: CS | After 1^st^ event: gradual resolution after 8 mo.  After 2^nd^ event: gradual improvement after wk. (12 yr.) | [12] |
| 14 | 11 | F | CD | At diagnosis | MRS, MRI and MOS dexter. MRM and MRI sinister | NON | CS | Rapid clinical and radiographic improvement after 2 mo. (2 mo.) | [13] |
| 15 | 44 | M | CD | + several yr. | MRS sinister | NON (4d prior stop CS) | AB and CS | No effect of AB. Rapid improvement with CS and remission after 3 mo. (3 mo.) | [14] |
| 16 | 54 | F | CD | - 2 yr. 5 mo., +3 yr. 6 mo., and +3 yr. 9 mo. | 1^st^ event: MRM sinister  2^nd^ event: NM  3^rd^ event: MRL dexter | 1^st^ event: NON  2^nd^ event: ICR and ‘diosmectite’  3^rd^ event: NON | 1^st^ event: NSAID and CS  2^nd^ event: CS  3^rd^ event: CS | 1^st^ event: resolution after 3 mo.  2^nd^ event: resolution after 1 mo.  3^rd^ event: resolution with no relapse over 1 yr. (7 yr.) | [15] |
| 17 | 32 | M | UC | +3 yr. | MRS sinister | NON | CS and ASA | Rapid improvement with no relapse over 1 yr. (4 yr.) | [16] |
| 18 | 53 | F | CD | +13 yr. and  +13 yr. 2 mo. | 1^st^ event: MRS and Mu. levator palpebrae superiores dexter  2^nd^ event: MRM sinister | 1^st^ event: sigmoidectomy and SSZ  2^nd^ event: relapse after tapering CS till 10mg/d | 1^st^ event: CS  2^nd^ event: CS, add-on AZA due to CS dependence (stop after 3wks. due to GI intolerance) and secondly IFX | Relapse with tapering CS after 2 mo.  Remission after start of IFX, no relapse over 2 yr. (15 yr.) | [17] |
| 19 | 40 | F | CD | +23 yr. | Bilateral MRI | prior therapy with CsA, 6-MP, and MTX | AB and CS | Complete resolution (NM) | [18] |
| 20 | 22 | F | CD | +4 yr. | Non-specified bilateral extra-ocular muscles | UST (prior AZA and IFX) | NSAID and CS | Complete resolution after 1 wk. (4 yr.) | [19] |
| 21 | 26 | F | CD | +3 yr. and  +3 yr. 4 mo. | 1^st^ and 2^nd^ event: MRM sinister | Vedo | 1^st^ event: CS  2^nd^ event: CS, add-on 6-MP | Rapid clinical and radiographic improvement with relapse after stop CS. Remission after start of 6-MP, no relapse over 4 mo. (4 yr.) | [20] |
| 22 | 38 | M | CD | +6 yr. | Masseter, Mu supraspinatus, Mu deltoideus, Mu pectoralis | NON | CS and AZA | Rapid improvement with no relapse of the polymyositis over 6 mo. (7 yr.) | [21] |
| 23 | 20 | M | CD | +7 yr. 5 mo. and  +7 yr. 7 mo. | 1^st^ event: MRL dexter  2^nd^ event: MRL sinister | 1^st^ event: ICR (prior AZA)  2^nd^ event: relapse after tapering CS till 5mg/d | 1^st^ event: CS  2^nd^ event: CS, add-on SSZ | Resolution after 1 wk., but relapse with tapering CS after 2 mo.  Remission after start of SSZ, no relapse over 4 mo. (8 yr.) | [22] |
| 24 | 45 | F | CD | +25 yr. | MRM dexter | ICR and redo-ICR (prior ASA) | AB and CS, due to intolerance of CS add-on AZA | Improvement after 2 d, with complete resolution after 9 mo. (26 yr.) | [23] |
| 25 | 37 | F | CD | +15 yr.,  +15 yr. 9 mo.,  +16yr. 5 mo., and  +16yr. 9 mo. | 1^st^, 2^nd^, 3^rd^ event: MRL dexter  4^th^ event: MRM dexter | 1^st^ event: ICR (prior MTX and IFX)  2^nd^ event: relapse after stop CS  3^rd^ and 4^th^ event: MTX | 1^st^ event: AB and CS  2^nd^ event: CS, add-on MTX due to arthritis  3^rd^ event: CS  4^th^ event: CS and switch to MMF | Resolution after 1 wk., but relapse with stop CS after 9 mo., 8 mo., and 4 mo.  Remission after start of MMF, no relapse over 3 mo. (17 yr.) | [23] |
| 26 | 15 | M | CD | -7 mo. (multiple events), and at diagnosis. | 1^st^ events: orbital muscles  2^nd^ event: bilateral MOS and all Mu. recti (not MRL sinister) | 1^st^ event: NON  2^nd^ event: AB and CS | 1^st^ events: CS, AB or self-resolving (6 ≠ episodes)  2^nd^ event: CS and MTX | Partially response after 1 wk. of high dose of CS during 2^nd^ event. Remission after start of MTX, no relapse over 8 mo. (1 yr.) | [23] |
| 27 | 57 | M | CD | NM | 1^st^ and 2^nd^ event: bilateral MRL + MRM sinister | 1^st^ event: UST since 6 wk. (prior: IFX)  2^nd^ event: UST | 1^st^ event: CS  2^nd^ event: CS and switch to Vedo | Initial resolution, but relapse with tapering CS.  Remission after start of Vedo, no relapse over 3 mo. (NM) | [24] |
| 28 | 35 | M | CD | +7 yr. | MRM dexter | ICR (prior: AZA, ASA, and recently stop IFX (-13wk.) and 6-MP (-6 wk.) due to side-effects | CS, ADA, and MTX | Rapid improvement after 48h. with no relapse over 10 mo. (8 yr.) | [25] |
| 29 | 14 | F | CD | -8 mo., -2 mo. and at diagnosis | 1^st^ event: left eye involvement  2^nd^ and 3^rd^ event: bilateral MRM and MRL | 1^st^ event: NON  2^nd^ event: NON  3^rd^ event: relapse after tapering CS till 10mg/d | 1^st^ event: AB  2^nd^ event: CS  3^rd^ event: CS and ICR | 2^nd^ event: rapid resolution after 48h, but relapse with tapering CS after 2 mo. 3^rd^ event: minimal improvement after CS. Only remission 1 mo. after ICR, with no relapse over 3 mo. (1 yr.) | [26] |
| 30 | 48 | F | CD | - 9 yr., -3 yr., ≠ events from -2 yr. and diagnosis,  +5 yr., and ≠ events from +5 and +9 yr. | 1^st^ event: MRI dexter  All other events: NM | 1^st^ event: NON  2^nd^ event: NON  3^rd^ episodes before diagnosis: NON  4^th^ event: recent stop SSZ  5^th^ episodes after diagnosis: SSZ | 1^st^ event: NON  2^nd^ event: CS  3^rd^ episodes before diagnosis: CS  4^th^ event: CS and restart SSZ  5^th^ episodes after diagnosis: CS | Gradual resolution over mo. after 1^st^ event. Rapid improvement after CS with complete resolution in all events afterwards, but with yearly relapse(s). Only after initial start of SSZ after diagnosis of CD 5 yrs. without relapse. (18 yr.) | [27] |
| 31 | 38 | F | CD | NM | NM | NM | CS and SSZ | NM | [28] |
| 32 | 17 | F | CD | At diagnosis | MRS | NM | NM | NM | [29] |
| 33 | 54 | F | CD | NM | Orbital | NM | CS and ICR | Two recurrent episodes (12mo.) | [30] |
| 34 | NM | NM | CD | NM | Orbital | NM | NM | NM | [31] |

**Legends:** Clinical characteristics of IBD patients associated with myositis of the head-neck region as an extra-intestinal manifestation. This table describes the age at last presentation of the myositis, disease localization of the myositis, as well as the time between IBD diagnosis and the diagnosis of the myositis (-: before, at diagnosis, +: after IBD diagnosis), current and past treatment use at time of diagnosis of the myositis, specific treatment for the myositis, and the outcome of the myositis treatment.

**Abbreviations:** AB: antibiotics; ADA: adalimumab; ASA: 5-aminosalicylic acid; AZA: azathioprine; CD: Crohn’s disease; CP: cyclophosphamide; CS: corticosteroids; CsA: ciclosporin; d: day; F: female; GI: gastrointestinal; h.: hours; IBD: inflammatory bowel disease; ICR: Ileocolic resection; IFX: infliximab; M: male; MMF: mycophenolate mofetil; mo.: months; MOS: musculus obliquus superior; 6-MP: 6-mercaptopurine; MRI: musculus rectus inferior; MRL: musculus rectus lateralis; MRM: musculus rectus medialis; MRS: musculus rectus superior; MTX: methotrexate; Mu.: musculus; NM: not mentioned; No.: number; NON: no therapy; NSAID: nonsteroidal anti-inflammatory drugs; SSZ: sulfasalazine; UC: ulcerative colitis; UST: ustekinumab; Vedo: Vedolizumab; wk.: week; yr.: years.

**References**:

1. Bennion J, Harris MA, Sivak-Callcott JA, Nguyen J. Bilateral diffuse orbital myositis in a patient with relapsing ulcerative colitis. *Ophthalmic Plast Reconstr Surg.* 2012;28:119-20. Doi: [10.1097/IOP.0b013e318244a34e](https://doi.org/10.1097/iop.0b013e318244a34e)

2. Bourikas LA, Roussomoustakaki M, Papadaki E, Valatas V, Koutroubakis IE, Papadakis KA. A case of orbital myositis preceding the intestinal symptoms of Crohn's disease. *J Crohns Colitis.* 2010;4:349-50. doi: [10.1016/j.crohns.2010.05.005](https://doi.org/10.1016/j.crohns.2010.05.005)

3. Camfield PR, White M, Warner HA, Lythgoe C. Orbital pseudotumor and Crohn disease. *J Pediatr.*1982;101:157-8. doi: [10.1016/s0022-3476(82)80214-x](https://doi.org/10.1016/s0022-3476(82)80214-x)

4. Cheng S, Vu P. Recurrent orbital myositis with radiological feature mimicking thyroid eye disease in a patient with Crohn's disease. *Orbit.* 2009;28:368-70. doi: [10.3109/01676830903104751](https://doi.org/10.3109/01676830903104751)

5. Culver EL, Salmon JF, Frith P, Travis SP. Recurrent posterior scleritis and orbital myositis as extra-intestinal manifestations of Crohn's disease: Case report and systematic literature review. *J Crohns Colitis.* 2008;2:337-42. doi: [10.1016/j.crohns.2008.06.002](https://doi.org/10.1016/j.crohns.2008.06.002)

6. Durno CA, Ehrlich R, Taylor R, Buncic JR, Hughes P, Griffiths AM. Keeping an eye on Crohn's disease: orbital myositis as the presenting symptom. *Can J Gastroenterol.* 1997;11:497-500. doi: [10.1155/1997/974282](https://doi.org/10.1155/1997/974282)

7. Garrity JA, Coleman AW, Matteson EL, Eggenberger ER, Waitzman DM. Treatment of recalcitrant idiopathic orbital inflammation (chronic orbital myositis) with infliximab. *Am J Ophthalmol.* 2004;138:925-30. doi: [10.1016/j.ajo.2004.06.077](https://doi.org/10.1016/j.ajo.2004.06.077)

8. Hernández-Garfella ML, Gracia-García A, Cervera-Taulet E, García-Villanueva C, Montero-Hernández J. Adalimumab for recurrent orbital myositis in Crohn's disease: report of a case with a 3-year follow-up. *J Crohns Colitis.* 2011;5:265-6. doi: [10.1016/j.crohns.2011.02.010](https://doi.org/10.1016/j.crohns.2011.02.010)

9. Hoilat GJ, Subedi A, Ayas MF, Ozden N. Keep an Eye Out for Crohn's Disease: Orbital myositis as the initial sign before gastrointestinal manifestations. *Eur J Case Rep Intern Med.* 2020;7:001964. doi: [10.12890/2020_001964](https://doi.org/10.12890/2020_001964)

10. Ishihara R, Jain SF, Perry D, Reinhardt A, Suh D, Legge R. Orbital pseudotumor as the presenting symptom of Crohn's disease in a male child. *Am J Ophthalmol Case Rep.* 2020;18:100669. doi: [10.1016/j.ajoc.2020.100669](https://doi.org/10.1016/j.ajoc.2020.100669)

11. Ishiwada N, Nagatake E, McParland Y, Hattori M, Tanabe M, Ohnuma N. Fulminant ulcerative colitis associated with both masseter muscle myositis and immunoglobulin M nephropathy. *Pediatr Int.* 1999;41:385-8. doi: [10.1046/j.1442-200x.1999.01076.x](https://doi.org/10.1046/j.1442-200x.1999.01076.x)

12. Jain S, Gottlob I. Orbital myositis associated with ulcerative colitis. *Am J Gastroenterol.* 2001;96:3442-4. doi: [10.1111/j.1572-0241.2001.05379.x](https://doi.org/10.1111/j.1572-0241.2001.05379.x)

13. Kondolot M, Unal E, Poyrazoglu G, Kara A, Yikilmaz A, Kumandas S. Orbital myositis associated with focal active colitis in a teenage girl. *Childs Nerv Syst.* 2012;28:641-3. doi: [10.1007/s00381-011-1613-8](https://doi.org/10.1007/s00381-011-1613-8)

14. Leibovitch I, Galanopoulos A, Selva D. Suppurative granulomatous myositis of an extra-ocular muscle in Crohn's disease. *Am J Gastroenterol.* 2005;100:2136-7. doi: [10.1111/j.1572-0241.2005.50395_10.x](https://doi.org/10.1111/j.1572-0241.2005.50395_10.x)

15. Maalouf T, Angioï K, George JL. Recurrent orbital myositis and Crohn's disease. *Orbit.* 2001;20:75-80. doi: [10.1076/orbi.20.1.75.2642](https://doi.org/10.1076/orbi.20.1.75.2642)

16. Macarez R, Bazin S, Weber F, Giordano P, Bernard P, Grubain S. Myosite orbitaire et rectocolite ulcéro-hémorragique [Orbital myositis associated with ulcerative colitis]. *J Fr Ophtalmol.* 2005;28:610-3. doi: [10.1016/s0181-5512(05)81103-9](https://doi.org/10.1016/s0181-5512(05)81103-9)

17. Pimentel R, Lago P, Pedroto I. Recurrent orbital myositis as an extra-intestinal manifestation of Crohn's disease. *J Crohns Colitis.* 2012;6:958-9. doi: [10.1016/j.crohns.2012.05.018](https://doi.org/10.1016/j.crohns.2012.05.018)

18. Ramalho J, Castillo M. Imaging of orbital myositis in Crohn's disease. *Clin Imaging.* 2008;32:227-9. doi: [10.1016/j.clinimag.2007.09.012](https://doi.org/10.1016/j.clinimag.2007.09.012)

19. Robertson IR, Pak KC, Harvey MM, Topping KL, Wilkerson RC, Cheatham JG. A Rare case of bilateral, sequential orbital myositis as an extra-intestinal manifestation of crohn’s disease. *Clin Case Rep J.* 2022;3:1–4.

20. Sandhu S, Wang T, Donet JA. Orbital myositis in a patient with ileal Crohn's disease in remission on vedolizumab. *ACG Case Rep J.* 2022;9:e00775. doi: [10.14309/crj.0000000000000775](https://doi.org/10.14309/crj.0000000000000775)

21. Seibold F, Klein R, Jakob F. Polymyositis, alopecia universalis, and primary sclerosing cholangitis in a patient with Crohn's disease. *J Clin Gastroenterol.* 1996;23:121-4. doi: [10.1097/00004836-199609000-00011](https://doi.org/10.1097/00004836-199609000-00011)

22. Squires RH Jr, Zwiener RJ, Kennedy RH. Orbital myositis and Crohn's disease. *J Pediatr Gastroenterol Nutr.* 1992;15:448-51. doi: [10.1097/00005176-199211000-00015](https://doi.org/10.1097/00005176-199211000-00015)

23. Vargason CW, Mawn LA. Orbital myositis as both a presenting and associated extraintestinal sign of Crohn's disease. *Ophthalmic Plast Reconstr Surg. 2017;33:S158-60.* doi: [10.1097/IOP.0000000000000475](https://doi.org/10.1097/iop.0000000000000475)

24. Vasudevan A, Tooley AA, Ida CM. Orbital Inflammation in an adult with Crohn disease. *JAMA Ophthalmol.* 2022;140:426-7. doi: [10.1001/jamaophthalmol.2021.4676](https://doi.org/10.1001/jamaophthalmol.2021.4676)

25. Verma S, Kroeker KI, Fedorak RN. Adalimumab for orbital myositis in a patient with Crohn's disease who discontinued infliximab: a case report and review of the literature. *BMC Gastroenterol.* 2013;13:59. doi: [10.1186/1471-230X-13-59](https://doi.org/10.1186/1471-230x-13-59)

26. Young RS, Hodes BL, Cruse RP, Koch KL, Garovoy MR. Orbital pseudotumor and Crohn disease. *J Pediatr.* 1981;99:250-2. doi: [10.1016/s0022-3476(81)80466-0](https://doi.org/10.1016/s0022-3476(81)80466-0)

27. Zenone T. Orbital myositis and Crohn's disease. *Int J Rheum Dis.* 2014;17:481-2. doi: [10.1111/1756-185X.12238](https://doi.org/10.1111/1756-185x.12238)

28. Verbraeken H, Ryckaert S, Demets W. Pseudotumor of the orbit and Crohn's disease. *Bull Soc Belge Ophtalmol.* 1984;210:65-72.

29. Weinstein JM, Koch K, Lane S. Orbital pseudotumor in Crohn's colitis. *Ann Ophthalmol.* 1984;16:275-8.

30. Smith JW. Orbital pseudotumour and Crohn's disease. *Am J Gastroenterol.* 1992;87:405-6.

31. Greenstein AJ, Janowitz HD, Sachar DB. The extra-intestinal complications of Crohn's disease and ulcerative colitis. A study of 700 patients. *Medicine (Baltimore).* 1976;55:401-12. doi: [10.1097/00005792-197609000-00004](https://doi.org/10.1097/00005792-197609000-00004)
